# The prognostic value of deep earlobe creases in patients with acute ischemic stroke

**DOI:** 10.3389/fcvm.2023.1096044

**Published:** 2023-05-30

**Authors:** Jiaping Xu, Lixuan Wang, Chunqing Zhang, Jiayun Wang, Danni Zheng, Yaqian Huang, Xia Zhang, Shoujiang You, Yongjun Cao, Chun-Feng Liu

**Affiliations:** ^1^ Department of Neurology and Suzhou Clinical Research Center of Neurological Disease, The Second Affiliated Hospital of Soochow University, Suzhou, China; ^2^ Department of Neurology, Honghui Hospital, Xi’an Jiaotong University, Xi’an, China; ^3^ Department of Neurology, Qinghai University Affiliated Hospital, Xining, China; ^4^ The George Institute for Global Health, Faculty of Medicine, University of New South Wales, Sydney, NSW, Australia; ^5^ Institutes of Neuroscience, Soochow University, Suzhou, China

**Keywords:** acute ischemic stroke, earlobe crease, prognostic value, frequency, characteristics

## Abstract

**Background and purpose:**

Data on earlobe crease (ELC) among patients with acute ischemic stroke (AIS) are limited. Here, we determined the frequency and characteristics of ELC and the prognostic effect of ELC among AIS patients.

**Methods:**

A total of 936 patients with acute AIS were enrolled during the period between December 2018 and December 2019. The patients were divided into those without and with ELC, unilateral and bilateral ELC, and shallow and deep ELC, according to the photographs taken of the bilateral ears. Logistic regression models were used to estimate the effect of ELC, bilateral ELC, and deep ELC on poor functional outcomes at 90 days (a modified Rankin Scale score ≥2) in AIS patients.

**Results:**

Among the 936 AIS patients, there were 746 (79.7%) patients with ELC. Among patients with ELC, there were 156 (20.9%) patients with unilateral ELC and 590 (79.1%) with bilateral ELC and 476 (63.8%) patients with shallow ELC and 270 (36.2%) with deep ELC. After adjusting for age, sex, baseline NIHSS score, and other potential covariates, patients with deep ELC were associated with a 1.87-fold [odds ratio (OR) 1.87; 95% confidence interval (CI), 1.13–3.09] and 1.63-fold (OR 1.63; 95%CI, 1.14–2.34) increase in the risk of poor functional outcome at 90 days in comparison with those without ELC or shallow ELC.

**Conclusion:**

ELC was a common phenomenon, and eight out of ten AIS patients had ELC. Most patients had bilateral ELC, and more than one-third had deep ELC. Deep ELC was independently associated with an increased risk of poor functional outcome at 90 days.

## Introduction

Earlobe crease (ELC) or Frank's sign is a wrinkle-like line extending diagonally from the tragus and across the lobule to the rear edge of the auricle of the ear, which Sanders T. Frank first described in 1973 (1). Subsequent studies indicated that ELC was one of the commonly visible age-related signs, and its occurrence increased with advancing age (2–4). Although ELC was observed historically, as shown in ancient Roman sculptures and Renaissance paintings, it has been recognized as a marker of cardiovascular pathology only in recent decades (5, 6).

Several observational studies indicated that ELC was more frequent in patients with coronary artery disease (CAD) (7–10) and peripheral arterial disease (PAD) (11, 12) and was reported to be independently associated with CAD and atherosclerosis-related diseases (7–15). Moreover, some studies also found that ELC was significantly associated with carotid artery intima-media thickness (CCA-IMT), plaque number (PN), vascular dysfunction, and metabolic syndrome (3, 14, 16).

Up to now, two studies from the US and Israel have observed that 59% and 77.8% of patients had ELC among 116 and 178 acute ischemic stroke (AIS) patients (17, 18). These findings from limited studies suggested a high rate of ELC in AIS patients and warrant further studies with large sample sizes from different regions to provide more information and evidence. Moreover, given the associations between ELC and age, vascular risk factors and atherosclerosis-related diseases, we hypothesize that ELC may exert prognostic utility on the clinical outcomes of AIS patients.

In the present study, we aim to investigate the frequency and characteristics of ELC to assess the relationship between ELC and clinical outcomes after the occurrence of AIS in a large sample of patients from China.

## Methods

### Study participants

We recruited patients with AIS between December 2018 and December 2019 at The Second Affiliated Hospital of Soochow University. A diagnosis of ischemic stroke was made according to criteria defined by the World Health Organization based on patient history, clinical data, and neuroimaging results [computed tomography (CT) or magnetic resonance imaging (MRI)]. A team of investigators reviewed the eligibility of study participants. Additional exclusion criteria were: (1) lack of photographs of the ears; (2) time from onset to admission more than 7 days. Finally, 936 patients were found to be potentially eligible for this analysis. (Flowchart of participant selection; Supplementary Figure S1).

### Data collection

Baseline information was collected for the following: patient demographics, vascular risk factors, stroke severity (as measured by the National Institutes of Health Stroke Scale, NIHSS; and the modified Rankin Scale, mRS), medication use, imaging data (ischemic location), Trial of Org 1,0172 in Acute Stroke Treatment (TOAST) classification, treatment (including intravenous thrombolytic and endovascular thrombectomy), and other diagnosis-related information. Vascular risk factors included a history of the following: hypertension, diabetes mellitus, ischemic stroke, intracerebral hemorrhage, atrial fibrillation, and coronary heart disease, and also included current smoking status and alcohol consumption. Information on the aforementioned factors was obtained through interviews with patients or their family members. Current smoking status was defined as having smoked at least one cigarette per day for the previous year or more. Alcohol consumption was defined as having consumed at least one alcoholic drink per day during the previous year. Hypertension was defined as having a systolic blood pressure (BP) ≥140 mmHg and/or diastolic BP ≥90 mmHg or using antihypertensive medications. Diabetes mellitus was defined as having fasting glucose ≥7.0 mmol/L (126 mg/dl), non-fasting glucose ≥11.1 mmol/L (200 mg/dl) with classic symptoms of hyperglycemia or hyperglycemic crisis, or using glucose-lowering drugs. Atrial fibrillation was defined as having a history of atrial fibrillation, confirmed by ≥1 electrocardiogram, or the presence of arrhythmia during hospitalization.

### Follow-up and outcome assessment

The study outcome was evaluated by using the mRS, obtained at 3 months by in-person or telephone interviews. The primary outcome was poor functional outcome at 3 months, defined as mRS ≥ 2. The second outcome was physical function across all seven levels of the mRS by ordinal analysis.

### The definition and different types of ELC

**Figure 1:**
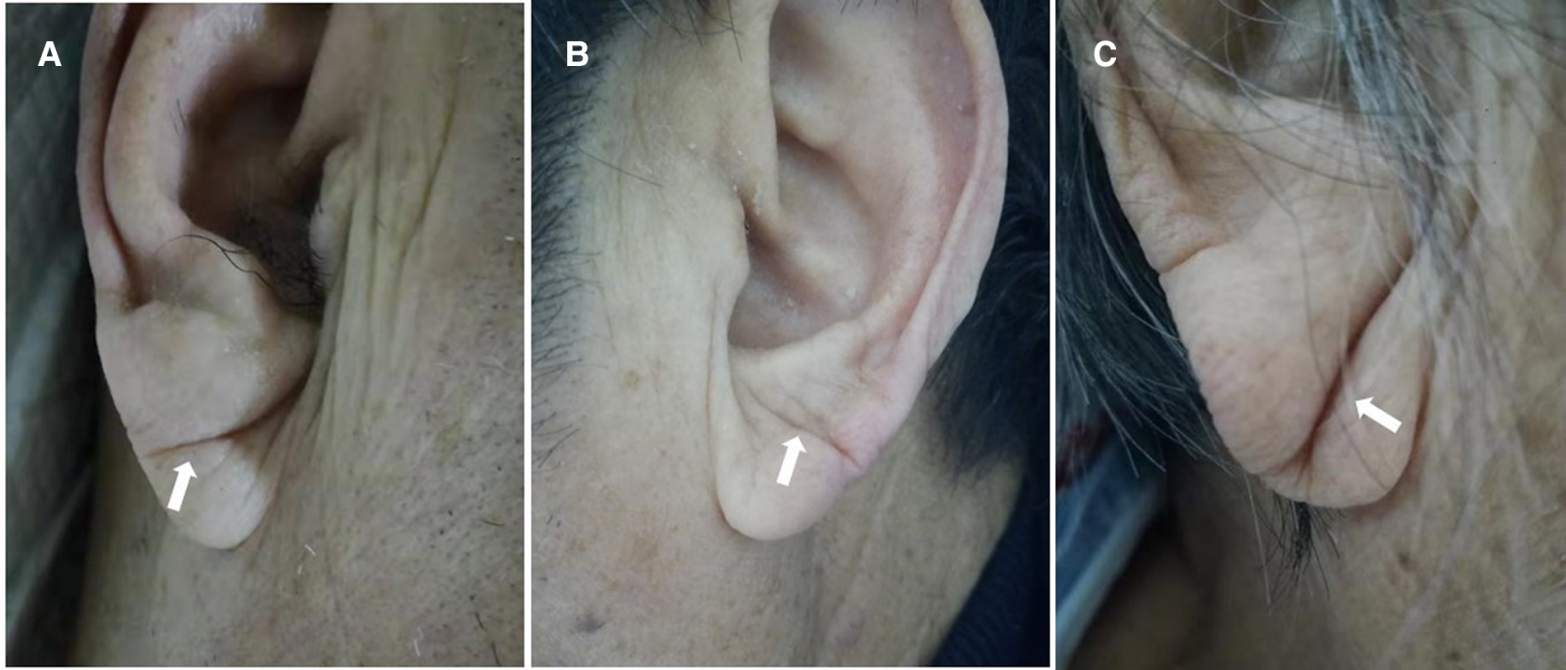
The photographs of typical ELC, including shallow and deep ELC. Typical ELC (**A**), shallow ELC (**B**) and deep ELC (**C**).

We took photographs of the bilateral ears of each patient. ELC was defined as a wrinkle-like line extending diagonally from the tragus and across the lobule to the rear edge of the auricle of the ear. A typical ELC is shown in Figure 1A (1). We divided patients with ELC into those who had unilateral vs. bilateral ELC and shallow vs. deep ELC (Figures 1B,C). ELC was considered shallow when the bilateral clear end of the fold could be seen. ELC was considered deep when the unilateral or bilateral end of the fold could not be seen (10).

### Statistical analysis

Continuous variables were expressed as mean ± standard deviation (SD) or median [interquartile range (IQR)] and were compared using the analysis of an independent Student's *t*-test or variance or the Wilcoxon rank-sum test. Categorical variables were expressed as a frequency (%) and were compared using the chi-square test.

Crude and multivariable logistic regression models were used to estimate the risk of poor functional outcomes at 3 months. Odds ratios (ORs) and 95% confidence intervals (CIs) were calculated for patients with ELC and for different types of ELC, with patients without ELC used as reference. The multivariable ordinal logistic regression was used to compare ordinal scores on the modified Rankin scale at 90 days among different groups. The potential confounders that were adjusted in the multivariable models were sex, baseline systolic BP, baseline diastolic BP, cigarette smoking status, alcohol drinking, history of hypertension, history of diabetes mellitus, history of ischemic stroke, history of intracerebral hemorrhage, history of atrial fibrillation, age (<45 years old vs. ≥45 years old), baseline NIHSS score (<3 vs. ≥3), intravenous thrombolytic, endovascular thrombectomy, and TOAST classification. All *P*-values were two-tailed, and a significance level of 0.05 was used. All analyses were conducted using the SPSS Version 17.0 statistical software.

## Results

A total of 936 AIS patients with complete data on conventional risk factors and photographs of bilateral ears were enrolled. The mean age was 67.8 years (±12.9), and the median NIHSS score was 3.0 (IQR, 1.0–6.0). Among these 936 AIS patients, 746 (79.7%) patients had ELC and 190 (20.3%) did not have ELC. In patients with ELC, there were 156 (20.9%) with unilateral ELC and 590 (79.1%) with bilateral ELC. There were 476 (63.8%) patients with shallow ELC and 270 (36.2%) with deep ELC. The baseline characteristics between patients with and without ELC are presented in Table 1. Compared with participants without ELC, those with ELC were more likely to be older and female, were less likely to smoke and consume alcohol, had a lower baseline diastolic BP, and had a more severe stroke (higher NIHSS) and other comorbidities such as hypertension, ischemic stroke, and atrial fibrillation. ELC patients also differed in terms of metabolic profile [higher high-density lipoprotein cholesterol (HDL-C), lower serum triglycerides, and lymphocyte count (Table 1)]. The baseline characteristics among patients without ELC and those with shallow and deep ELC are presented in Supplementary Table S1. Compared with patients without ELC, those with deep ELC were more likely to be older and female, were less likely to smoke and consume alcohol, and more likely to have a history of ischemic stroke, atrial fibrillation, and undergone endovascular thrombectomy treatment more times. Deep ELC patients also had more severe stroke, higher HDL-C and C-reactive protein levels, and lower baseline diastolic BP, serum triglycerides, and lymphocyte count (Supplementary Table S1). The baseline characteristics among patients without ELC and unilateral and bilateral ELC groups are presented in Supplementary Table S2.

**Table 1:** Baseline characteristics between patients with and without earlobe crease (ELC).

Characteristics^a^	With ELC	Without ELC	*P*-value
Number of subjects	746	190	
Demographics			
Age, years	70.8 ± 10.5	56.1 ± 14.5	<0.001
Male sex	453 (60.7)	136 (71.6)	0.006
Cigarette smoking status	250 (33.5)	85 (44.7)	0.004
Alcohol consumption	172 (23.1)	65 (34.2)	0.002
Clinical features			
Time from onset to hospital, h	12.0 (6.0–48.0)	12.0 (8.0–48.0)	0.207
Baseline systolic BP, mm Hg	151.8 ± 23.4	150.0 ± 25.6	0.370
Baseline diastolic BP, mm Hg	82.4 ± 13.2	87.7 ± 16.0	<0.001
TG, mmol/L	1.2 (0.9–1.7)	1.4 (1.0–1.9)	0.020
TC, mmol/L	4.5 (3.9–5.2)	4.6 (4.0–5.3)	0.223
LDL-C, mmol/L	2.7 (2.1–3.3)	2.8 (2.1–3.4)	0.160
HDL-C, mmol/L	1.1 (0.9–1.3)	1.0 (0.9–1.3)	0.021
FPG, mmol/L	5.7 (5.0–7.1)	5.5 (5.0–7.1)	0.246
WBC, 10^3^/µl	7.1 (5.7–8.8)	7.3 (5.6–9.0)	0.794
Lymphocyte count, 10^3^/µl	1.5 (1.1–2.0)	1.7 (1.3–2.2)	0.002
CRP, mg/L	5.3 (4.7–6.0)	5.2 (4.7–5.8)	0.112
Fibrinogen, g/L	3.2 (2.6–3.8)	3.1 (2.6–3.6)	0.264
Baseline NIHSS score	3.0 (1.0–7.0)	2.0 (1.0–4.0)	<0.001
Intravenous thrombolytic	99 (13.3)	18 (9.5)	0.158
Endovascular thrombectomy	47 (6.3)	8 (4.2)	0.274
Medical history			
History of hypertension	597 (80.0)	125 (65.8)	<0.001
History of diabetes mellitus	253 (33.9)	62 (32.6)	0.738
History of coronary heart disease	40 (5.4)	7 (3.7)	0.344
History of atrial fibrillation	132 (17.7)	16 (8.4)	0.002
History of ischemic stroke	204 (27.3)	28 (14.7)	<0.001
History of ICH	17 (2.3)	4 (2.1)	0.884
Medication history			
Antihypertensive therapy	471 (63.1)	79 (41.6)	<0.001
Antiplatelet therapy	125 (16.8)	19 (10.0)	0.021
Anticoagulation therapy	21 (2.8)	4 (2.1)	0.577
Antiglycemic therapy	175 (23.5)	30 (15.8)	0.022
Statin therapy	88 (11.8)	13 (6.8)	0.049
Ischemic location			0.643
Lobar	143 (19.2)	29 (15.3)	
Deep	255 (34.2)	70 (36.8)	
Lobar and deep	171 (22.9)	46 (24.2)	
Cerebellar and brainstem	177 (23.7)	45 (23.7)	
TOAST classification			<0.001
Large-artery atherosclerosis	460 (61.7)	112 (58.9)	
Cardioembolism	137 (18.4)	19 (10.0)	
Small-vessel occlusion	118 (15.8)	41 (21.6)	
Stroke of other determined etiology	27 (3.6)	13 (6.8)	
Stroke of undetermined etiology	4 (0.5)	5 (2.6)	

BP, blood pressure; TG, triglycerides; TC, total cholesterol; LDL-C, low-density lipoprotein cholesterol; HDL-C, high-density lipoprotein cholesterol; FPG, fasting plasma glucose; NIHSS, National Institutes of Health Stroke Scale; TOAST, Trial of Org 10,172 in Acute Stroke Treatment; WBC, white blood cell; CRP, C-reactive protein; ICH, intracerebral hemorrhage.

^a^ Continuous variables are expressed as mean ± standard deviation or as median (interquartile range). Categorical variables are expressed as frequency (percent).

Among 936 AIS patients, 114 (12.2%) patients were lost to follow-up and 822 (87.8%) patients completed follow-up at 90 days. There were 395 (48.1%) patients with poor functional outcome at 90 days. In the unadjusted model, patients with ELC demonstrated a 2.03-fold increase in the odds of poor functional outcome (95% CI, 1.41–2.92; *P*-value < 0.001) (Table 2). However, the association between patients with ELC and increased poor functional outcome at 90 days was insignificant after adjusting for age, sex, history of ischemic stroke, baseline NIHSS score, TOAST classification, and other confounders (*P*-value = 0.213) (Table 2).

**Table 2:** Odds ratios and 95% confidence intervals for poor functional outcome at 90 days (mRS 2–6) in patients with and without ELC.

		Unadjusted		Model 1		Model 2	
	Cases (%)	OR (95% CI)	*P*-value	OR (95% CI)	*P*-value	OR (95% CI)	*P*-value
Without or with ELC			<0.001		0.015		0.213
Without ELC	53 (34.2)	1.00 (reference)		1.00 (reference)		1.00 (reference)	
With ELC	342 (51.3)	2.03 (1.41–2.92)		1.62 (1.10–2.40)		1.32 (0.85–2.03)	

ELC, earlobe crease; mRS, modified Rankin Scale; OR, odds ratio; CI, confidence interval.

Model 1, adjusted for sex, systolic BP, diastolic BP, cigarette smoking status, alcohol drinking, history of hypertension, history of diabetes mellitus, history of atrial fibrillation, history of ischemic stroke, and history of intracerebral hemorrhage.

Model 2, adjusted for sex, systolic BP, diastolic BP, cigarette smoking status, alcohol drinking, history of hypertension, history of diabetes mellitus, history of atrial fibrillation, history of ischemic stroke, history of intracerebral hemorrhage, age (<45 vs. ≥45 years old), baseline National Institute of Health Stroke Scale score (NIHSS<3 vs. NIHSS ≥ 3), intravenous thrombolytic, endovascular thrombectomy, and TOAST classification.

Next, we investigated the different types of ELC and 90-day functional outcomes in Table 3. Compared with patients without ELC, those with deep ELC and bilateral ELC had a 3.29-fold (95% CI, 2.15–5.02; *P*-trend < 0.001) and 2.05-fold (95% CI, 1.41–2.98; *P*-trend < 0.001) increased risk of poor functional outcome at 90 days in the unadjusted model (Table 3). After adjusting for age, sex, history of ischemic stroke, baseline NIHSS score, TOAST classification, and other confounders, the odds of poor outcome at 90 days were significantly higher among participants with deep ELC compared with those without ELC (OR, 1.87; 95% CI, 1.13–3.09; *P*-trend = 0.006) (Table 3 and Supplementary Figure S2A). Moreover, we found that patients with deep ELC were also at a significantly increased risk of having poor functional outcome compared with those with shallow ELC (OR, 1.63; 95% CI, 1.14–2.34; *P*-value = 0.008) in the multivariable model (Table 3 and Supplementary Figure S2B). However, the relationship between patients with bilateral ELC and poor functional outcome was not significant after adjusting for potential confounders (*P*-trend = 0.191) (Table 3 and Supplementary Figure S2C).

**Table 3:** Odds ratios and 95% confidence intervals for poor functional outcome at 90 days (mRS 2–6) in patients with different shapes of ELCs.

		Unadjusted		Model 1		Model 2	
	Cases (%)	OR (95% CI)	*P*-trend	OR (95% CI)	*P*-trend	OR (95% CI)	*P*-trend
Without, unilateral or bilateral ELC			<0.001		0.026		0.191
Without ELC	53 (34.2)	1.00 (reference)		1.00 (reference)		1.00 (reference)	
Unilateral ELC	71 (50.0)	1.93 (1.21–3.07)		1.58 (0.96–2.58)		1.22 (0.71–2.09)	
Bilateral ELC	271 (51.6)	2.05 (1.41–2.98)		1.64 (1.10–2.44)		1.35 (0.86–2.09)	
Without, shallow or deep ELC			<0.001		<0.001		0.006
Without ELC	53 (34.2)	1.00 (reference)		1.00 (reference)		1.00 (reference)	
Shallow ELC	190 (44.6)	1.55 (1.06–2.27)		1.35 (0.90–2.02)		1.13 (0.73–1.77)	
Deep ELC	152 (63.1)	3.29 (2.15–5.02)		2.45 (1.55–3.85)		1.87 (1.13–3.09)	
Shallow or deep ELC			<0.001		0.001		0.008
Shallow ELC	190 (44.6)	1.00 (reference)		1.00 (reference)		1.00 (reference)	
Deep ELC	152 (63.1)	2.12 (1.53–2.93)		1.79 (1.27–2.52)		1.63 (1.14–2.34)	

ELC, earlobe crease; mRS, modified Rankin Scale; OR, odds ratio; CI, confidence interval.

Model 1, adjusted for sex, systolic BP, diastolic BP, cigarette smoking status, alcohol drinking, history of hypertension, history of diabetes mellitus, history of atrial fibrillation, history of ischemic stroke, and history of intracerebral hemorrhage.

Model 2, adjusted for sex, systolic BP, diastolic BP, cigarette smoking status, alcohol drinking, history of hypertension, history of diabetes mellitus, history of atrial fibrillation, history of ischemic stroke, history of intracerebral hemorrhage, age (<45 vs. ≥45 years old), baseline National Institute of Health Stroke Scale score (NIHSS < 3 vs. NIHSS ≥ 3), intravenous thrombolytic, endovascular thrombectomy, and TOAST classification.

**Figure 2:**
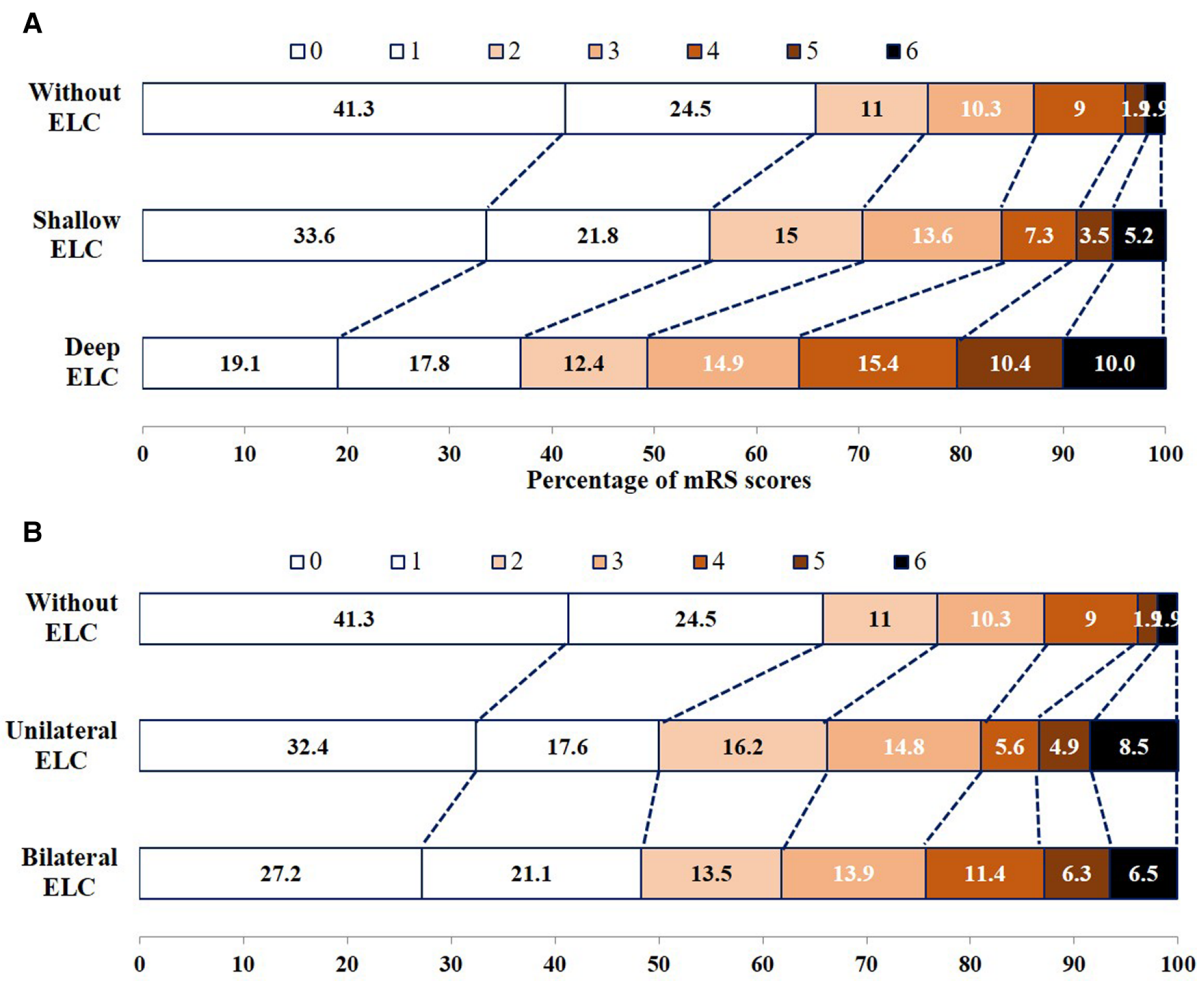
Functional outcomes at 90 days among patients without ELC, with shallow ELC and deep ELC (**A**), without ELC, and with unilateral ELC and bilateral ELC (**B**), according to score on the modified Rankin Scale.

Supplementary Figure S3 shows the relationship between neurological recovery between patients with and without ELC, and no significant increase in the score on the mRS was noted among patients with ELC in the multivariable ordinal logistic regression models (*P *= 0.703). The relationship among patients without ELC, with shallow and deep ELC, without ELC, and with unilateral and bilateral ELC in terms of neurological recovery is shown in Figures 2A,B. Multivariable ordinal logistic regression models showed that deep ELCs were significantly associated with higher mRS scores compared with no ELC (*P *= 0.007). Bilateral ELCs were not associated with higher mRS scores compared with no ELC (*P *= 0.296).

## Discussion

In this study, we reported the prevalence, characteristics, and prognostic effect of ELC among patients with AIS. ELC in AIS patients was a common phenomenon, occurring in nearly 80% of all patients. Most of these subjects had bilateral ELC and one in three patients had deep ELC. Moreover, we found that having deep ELC was independently associated with an increased risk of poor functional outcomes at 90 days compared with not having ELC or having shallow ELC.

ELC was first described by T. Frank in patients with CAD and was reported as a visible aging sign in the general population and in patients with different diseases (1–18). Population studies indicated that the frequency of ELC increased with age (2–4, 19). A cross-sectional study of 3,835 healthy subjects of South Korea noted that 21% of them had ELC, and the frequency of ELC increased from 9.7% to 44.1% with age ranging from 20 to 79 years (3). Several observational studies demonstrated that the rate of ELC was higher in patients with CAD and PAD than in healthy controls and ELC was independently associated with CAD, PAD, and other vascular factors such as hypertension and diabetes (7–15, 20). A meta-analysis of 37 studies covering more than 31,100 subjects indicated that 62% of patients with CAD had ELC, and the risk of CAD was 3.3-fold higher in patients with ELC than in those without ELC (20).

Data of ELC in patients with AIS were limited. Two studies of a total of 294 AIS patients indicated that 59% and 77.8% subjects with ELC and a high rate of hypertension and diabetes was seen in those with ELC (17, 18). In the present study of 936 AIS patients, we found 746 (79.7%) patients with ELC, which is consistent with the finding of previous studies that showed a high rate of ELC among AIS patients (17, 18). We also showed that patients with ELC were more likely to have a history of hypertension, atrial fibrillation, and ischemic stroke, which corroborates findings from earlier studies reporting an association between ELC and vascular factors.

Interestingly, some studies have focused on the sums and shapes of ELCs among patients with different diseases (9, 10, 16). Evidence from previous studies demonstrated that the rates of those having bilateral ELC were significantly higher than those having unilateral ELC (9, 10, 16). A study from Spanish patients with cardiovascular events first defined the shape of ELC according to its depth and found that about 9% of patients had deep ELC (10). In our study, we found that the rate of bilateral ELC was four times higher than that of unilateral ELC, which agrees with the findings on other diseases (9, 10, 16). Deep ELC was found in one-third of the individuals in our study, which is a higher frequency than that identified in a previous study among patients with cardiovascular events (10). Patients with deep ELC were more likely to be older, with the highest NIHSS scores and the highest percentage of prior comorbidities. The characteristics of ELC, especially the shape of ELC, need to be evaluated in future studies.

To our knowledge, this is the first study to prospectively explore the prognostic utility of ELC and functional outcome after AIS. In the unadjusted model, we found that patients with ELC, unilateral ELC, bilateral ELC, shallow ELC, and deep ELC were all associated with a higher risk of poor functional outcome at 90 days compared with those without ELC. After adjusting for potential confounders, only deep ELC was significantly associated with an increased risk of poor functional outcome. Patients with deep ELC had a 1.87-fold and 1.63-fold increase in the risk of poor functional outcome compared with those without ELC and with shallow ELC.

The precise pathophysiology of ELC is unclear. Poor blood supply in the arteries connecting the earlobes and microvascular disease with loss or degeneration of elastin fibers with rupture of the elastic fibers may be the underlying mechanisms (19, 21). Also, the causes of increased poor functional outcomes among AIS patients with deep ELC have not been completely understood. Several hypotheses have been proposed. First, a possible reason for the link between deep ELC and poor functional outcome is that ELC might reflect endothelial dysfunction (16) and metabolic syndrome (3), which are known predictors of poor outcome after AIS (22–24). Second, patients with ELC are reported to have a high carotid artery intima-media thickness, plaque number (14), and white matter hyperintensities (25), which may lead to poor outcomes after stroke (26, 27). Third, our study found that patients with deep ECL had the highest CRP levels and lowest lymphocyte counts, suggesting that deep ECL may reflect increased inﬂammation, which contributes to poor functional outcome after AIS (28, 29).

Our findings raise several important issues related to clinical practice. First, we found a high frequency of ELC in patients with ischemic stroke and described the characteristics of ELC in a relatively large dataset. Our data support the relationship between ELC, age-related signs, and ischemic stroke. Secondly, our study is the first to evaluate the prognostic effect of ELC on functional outcomes. We noted that deep ELC was significantly associated with poor functional outcomes at 90 days. Our findings suggest that deep ELC may be an easy-to-observe and widely used indicator for predicting the functional outcomes after ischemic stroke.

There are still some potential limitations in this study that merit consideration. Firstly, there may be selection bias in our study, as 114 (12.2%) patients who were lost to follow-up at 90 days were young in age with a relatively low NIHSS score and had a lower rate of ELC. Secondly, the scope for the generalizability of our study findings that were based on a single center may be limited. Thirdly, the follow-up time was short, and therefore, we could not evaluate the association between ELC and vascular events including recurrent stroke.

## Conclusion

In summary, our study has shown the high frequency and characteristics of ELC among patients with acute ischemic stroke. Deep ELC independently predicted poor functional outcome at 90 days after AIS. Our findings may assist clinicians in the early assessment of the prognostication of patients with AIS as ELC is an easily observed sign. Further prospective studies from other populations are needed to verify our findings and clarify the potential mechanisms.

## Data Availability

The data that support the findings of this study are available on request from the corresponding author.
