# Supplementary material for: The prognostic value of deep earlobe creases in patients with acute ischemic stroke
Source: Front Cardiovasc Med. 2023 May 30;10:1096044. doi: 10.3389/fcvm.2023.1096044 (PMC10266351; doi:10.3389/fcvm.2023.1096044)

ONLINE SUPPLEMENT

The prognostic of deep Earlobe crease in patients with acute ischemic stroke

Contents

Supplementary Figure 1. Patient flowchart.

Supplementary Table 1. Baseline characteristics between patients without ELC, with deep and shallow ELC.

Supplementary Table 2. Baseline characteristics between patients without ELC, with unilateral and bilateral ELC.

Supplementary Figure 2. Association between ELC and 90-day poor functional outcome. Compared among without ELC, with shallow ELC and deep ELC (2A), compared between with shallow ELC and deep ELC (2B), compared among without ELC, with unilateral ELC and bilateral ELC (2C).

Supplementary Figure 3. Functional outcomes at 90 days between patients with and without ELC, according to score on the Modified Rankin Scale.

Supplementary Figure 1. Patient flowchart.

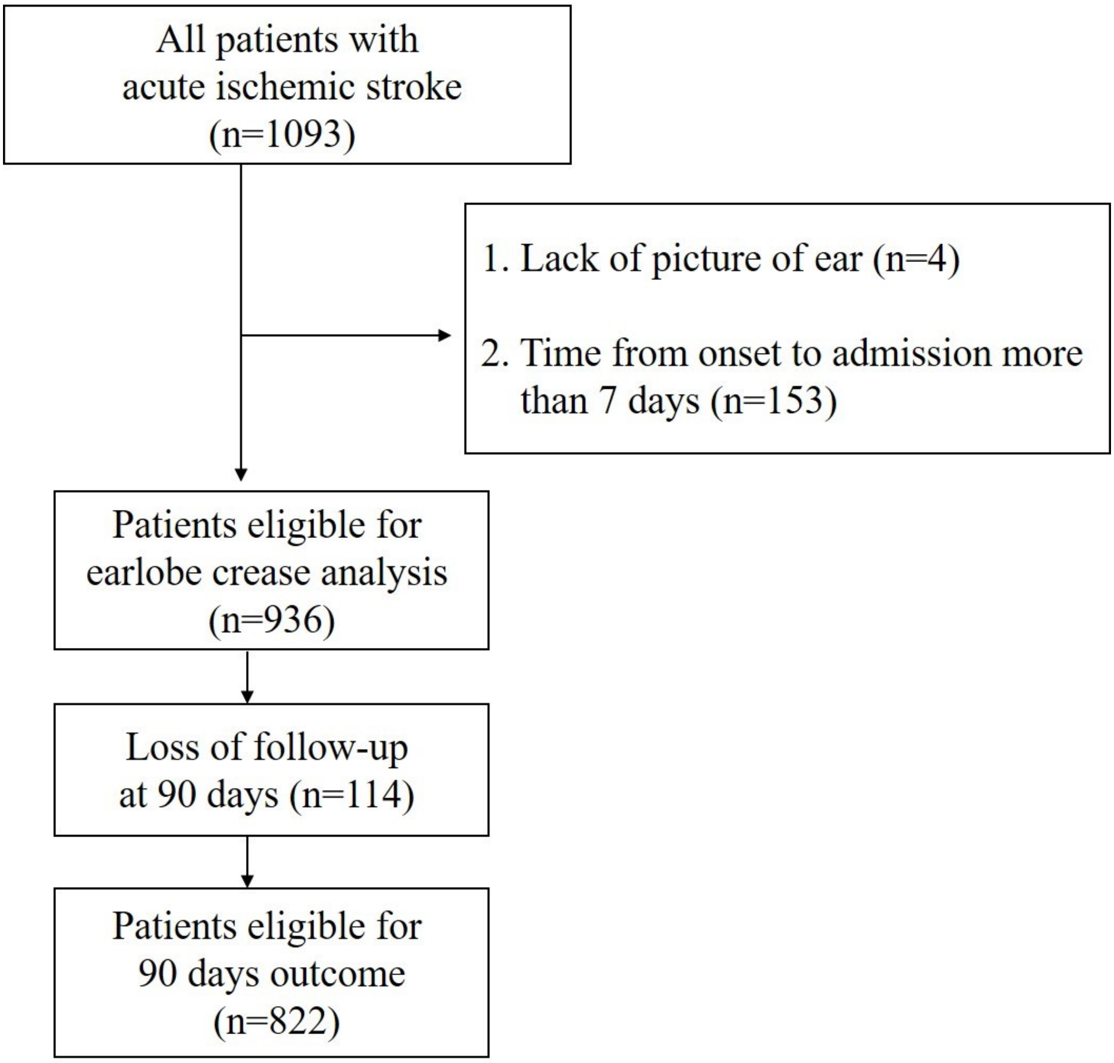

Supplementary Table 1. Baseline characteristics between patients without ELC, with deep and shallow ELC

Characteristics [a]	Without ELC	Shallow ELC	Deep ELC	P-value
Number of subjects	190	476	270	
Demographics				
Age, y	56.1±14.5	68.4±10.4	75.0±9.3	<0.001
Male sex	136 (71.6)	307 (64.5)	146 (54.1)	<0.001
Cigarette smoking status	85 (44.7)	172 (36.1)	78 (28.9)	0.002
Alcohol consumption	65 (34.2)	125 (26.3)	47 (17.4)	<0.001
Clinical features				
Time from onset to hospital, h	12.0 (8.0–48.0)	12.0 (6.0–48.0)	12.0 (5.0–48.0)	0.332
Baseline systolic BP, mm Hg	150.0±25.6	152.1±22.9	151.1±24.3	0.576
Baseline diastolic BP, mm Hg	87.7±16.0	83.2±13.0	80.9±13.4	<0.001
TG, mmol/L	1.4 (1.0–1.9)	1.3 (1.0–1.8)	1.1 (0.9–1.6)	<0.001
TC, mmol/L	4.6 (4.0–5.3)	4.5 (3.9–5.2)	4.5 (3.9–5.2)	0.449
LDL-C, mmol/L	2.8 (2.1–3.4)	2.7 (2.1–3.3)	2.6 (2.0–3.3)	0.216
HDL-C, mmol/L	1.0 (0.9–1.3)	1.1 (0.9–1.3)	1.2 (1.0–1.4)	<0.001
FPG, mmol/L	5.5 (5.0–7.1)	5.6 (5.0–6.9)	5.7(5.1–7.5)	0.376
WBC, 10^3/uL	7.3 (5.6–9.0)	7.1 (5.8–8.7)	7.1 (5.6–8.9)	0.934
Lymphocyte count, 10^3/uL	1.7 (1.3–2.2)	1.5 (1.1–2.1)	1.4 (0.9–1.9)	<0.001
CRP, mg/L	5.2 (4.7–5.8)	5.0 (4.7–5.8)	5.6 (4.7–6.7)	0.001
Fibrinogen, g/L	3.1 (2.6–3.6)	3.1 (2.6–3.7)	3.2 (2.7–3.8)	0.134
Baseline NIHSS score	2.0 (1.0–4.0)	2.0 (1.0–6.0)	4.0 (2.0–10.0)	<0.001
Intravenous thrombolytic	18 (9.5)	57 (12.0)	42 (15.6)	0.134
Endovascular thrombectomy	8 (4.2)	23 (4.8)	24 (8.9)	0.042
Medical history				
History of hypertension	125 (65.8)	373 (78.4)	224 (83.0)	<0.001
History of diabetes mellitus	62 (32.6)	158 (33.2)	95 (35.2)	0.812
History of coronary heart disease	7 (3.7)	20 (4.2)	20 (7.4)	0.100
History of atrial fibrillation	16 (8.4)	66 (13.9)	66 (24.4)	<0.001
History of ischemic stroke	28 (14.7)	113 (23.7)	91 (33.7)	<0.001
History of ICH	4 (2.1)	10 (2.1)	7 (2.6)	0.903
Medication history				
Antihypertensive therapy	79 (41.6)	291 (61.1)	180 (66.7)	<0.001
Antiplatelet therapy	19 (10.0)	65 (13.7)	60 (22.2)	0.001
Anticoagulation therapy	4 (2.1)	10 (2.1)	11 (4.1)	0.264
Antiglycemic therapy	30 (15.8)	105 (22.1)	70 (25.9)	0.035
Statin therapy	13 (6.8)	47 (9.9)	41 (15.2)	0.012
Ischemic location				0.065
Lobar	29 (15.3)	85 (17.9)	58 (21.5)	
Deep	70 (36.8)	179 (37.6)	76 (28.1)	
Lobar and deep	46 (24.2)	96 (20.2)	75 (27.8)	

Cerebellar and brainstem	45 (23.7)	116 (24.4)	61 (22.6)	
TOAST classification				<0.001
Large-artery atherosclerosis	112 (58.9)	299 (62.8)	161 (59.6)	
Cardioembolism	19 (10.0)	70 (14.7)	67 (24.8)	
Small-vessel occlusion	41 (21.6)	85 (17.9)	33 (12.2)	
Stroke of other determined etiology	13 (6.8)	19 (4.0)	8 (3.0)	
Stroke of undetermined etiology	5 (2.6)	3(0.6)	1(0.4)	

*Continuous variables are expressed as mean ± standard deviation or as median (interquartile range). Categorical variables are expressed as frequency (percent).

Abbreviations: BP, blood pressure; TG, triglycerides; TC, total cholesterol; LDL-C, low-density lipoprotein cholesterol; HDL-C, high-density lipoprotein cholesterol; FPG, fasting plasma glucose; NIHSS, National Institutes of Health Stroke Scale; TOAST, Trial of Org 10172 in Acute Stroke Treatment; WBC, white blood cell; CRP, C-reactive protein; ICH, intracerebral hemorrhage.

Supplementary Table 2. **Baseline characteristics between patients without ELC, with unilateral and bilateral ELC**

Characteristics [a]	Without ELC	Unilateral ELC	Bilateral ELC	*P*-value
Number of subjects	190	156	590	
Demographics				
Age, y	56.1±14.5	66.4±11.3	72.0±10.0	<0.001
Male sex	136 (71.6)	109 (69.9)	344 (58.3)	0.001
Cigarette smoking status	85 (44.7)	58 (37.2)	192 (32.5)	0.009
Alcohol consumption	65 (34.2)	38 (22.4)	134 (22.7)	0.006
Clinical features				
Time from onset to hospital, h	12.0 (8.0–48.0)	12.0 (5.0–18.5)	12.0 (6.0–48.0)	0.070
Baseline systolic BP, mm Hg	150.0±25.6	149.7±22.8	152.3±23.6	0.305
Baseline diastolic BP, mm Hg	87.7±16.0	83.1±11.8	82.2±13.5	<0.001
TG, mmol/L	1.4 (1.0–1.9)	1.2 (0.9–1.7)	1.2 (0.9–1.7)	0.066
TC, mmol/L	4.6 (4.0–5.3)	4.5 (4.0–5.3)	4.5 (3.9–5.2)	0.360
LDL-C, mmol/L	2.8 (2.1–3.4)	2.7 (2.2–3.4)	2.7 (2.0–3.3)	0.217
HDL-C, mmol/L	1.0 (0.9–1.3)	1.1 (0.9–1.3)	1.1 (0.9–1.3)	0.034
FPG, mmol/L	5.5 (5.0–7.1)	5.5 (5.1–6.6)	5.7 (5.0–7.2)	0.232
WBC, 10^3/uL	7.3 (5.6–9.0)	7.4 (5.8–9.0)	7.0 (5.7–8.8)	0.359
Lymphocyte count, 10^3/uL	1.7 (1.3–2.2)	1.6 (1.1–2.1)	1.5 (1.1–2.0)	0.003
CRP, mg/L	5.2 (4.7–5.8)	5.2 (4.6–5.8)	5.3 (4.7–6.1)	0.111
Fibrinogen, g/L	3.1 (2.6–3.6)	3.1 (2.6–3.6)	3.2 (2.6–3.8)	0.463
Baseline NIHSS score	2.0 (1.0–4.0)	3.0 (1.0–9.0)	3.0 (1.0–7.0)	<0.001
Intravenous thrombolytic	18 (9.5)	23 (14.7)	76 (12.9)	0.303
Endovascular thrombectomy	8 (4.2)	12 (7.7)	35 (5.9)	0.389
Medical history				
History of hypertension	125 (65.8)	123 (78.8)	474 (80.3)	<0.001
History of diabetes mellitus	62 (32.6)	47 (30.1)	206 (34.9)	0.502
History of coronary heart disease	7 (3.7)	9 (5.8)	31 (5.3)	0.618
History of atrial fibrillation	16 (8.4)	24 (15.4)	108 (18.3)	0.005
History of ischemic stroke	28 (14.7)	43 (27.6)	161 (27.3)	0.002
History of ICH	4 (2.1)	4 (2.6)	13 (2.2)	0.955
Medication history				
Antihypertensive therapy	79 (41.6)	100 (64.1)	371 (62.9)	<0.001
Antiplatelet therapy	19 (10.0)	20 (12.8)	105 (17.8)	0.022
Anticoagulation therapy	4 (2.1)	6 (3.8)	15 (2.5)	0.577
Antiglycemic therapy	30 (15.8)	32 (20.5)	143 (24.2)	0.045
Statin therapy	13 (6.8)	14 (9.0)	74 (12.5)	0.064
Ischemic location				0.770
Lobar	29 (15.3)	25 (16.0)	118 (20.0)	
Deep	70 (36.8)	56 (35.9)	199 (33.7)	
Lobar and deep	46 (24.2)	39 (25.0)	132 (22.4)	

Cerebellar and brainstem	45 (23.7)	36 (23.1)	141 (23.9)	
TOAST classification				0.002
Large-artery atherosclerosis	112 (58.9)	93 (59.6)	367 (62.2)	
Cardioembolism	19 (10.0)	26 (16.7)	111 (18.8)	
Small-vessel occlusion	41 (21.6)	26 (16.7)	92 (15.6)	
Stroke of other determined etiology	13 (6.8)	10 (6.4)	17 (2.9)	
Stroke of undetermined etiology	5 (2.6)	1(0.6)	3(0.5)	

*Continuous variables are expressed as mean ± standard deviation or as median (interquartile range). Categorical variables are expressed as frequency (percent).

Abbreviations: BP, blood pressure; TG, triglycerides; TC, total cholesterol; LDL-C, low-density lipoprotein cholesterol; HDL-C, high-density lipoprotein cholesterol; FPG, fasting plasma glucose; eGFR, estimated glomerular filtration rate; mRS, modified Rankin Scale; NIHSS, National Institutes of Health Stroke Scale; Q, quartile; TOAST, Trial of Org 10172 in Acute Stroke Treatment; WBC, white blood cell; CRP, C-reactive protein; ICH, intracerebral hemorrhage.

Supplementary Figure 2. Association between ELC and 90-day poor functional outcome. Compared among without ELC, with shallow ELC and deep ELC (2A), compared between with shallow ELC and deep ELC (2B), compared among without ELC, with unilateral ELC and bilateral ELC (2C).

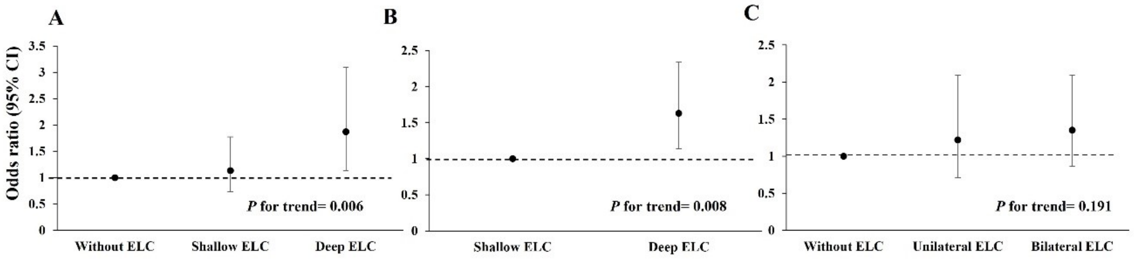

Supplementary Figure 3. Functional outcomes at 90 days between patients with and without ELC, according to score on the Modified Rankin Scale.

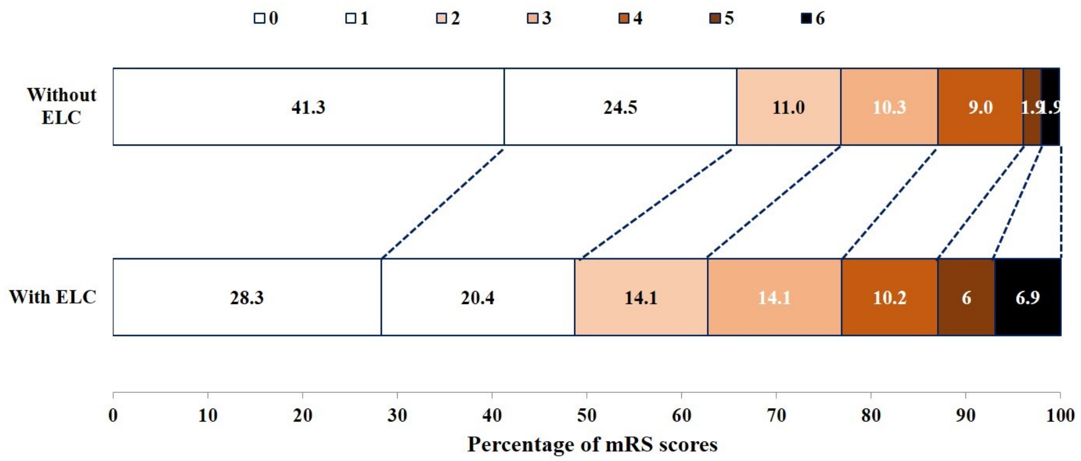

Supplement: Supplementary file 1 [file Presentation1.pdf]
